# Tsp1 promotes alveolar stem cell proliferation and its down-regulation relates to lung inflammation in intralobar pulmonary sequestration

**DOI:** 10.18632/oncotarget.19952

**Published:** 2017-08-04

**Authors:** Kuan Li, Qi Wu, Xin Sun, Yan Geng, Dong Leng, Hongwei Li, Subei Zhang, Qiaoxing Wang, Junping Wu, Long Xu, Xue Li, Yu Li, Qiuyang Zhang, Adrianne Kurkciyan, Jiurong Liang, Dianhua Jiang, Huaiyong Chen

**Affiliations:** ^1^ Department of Basic Medicine, Haihe Clinic College of Tianjin Medical University, Tianjin, China; ^2^ Key Research Laboratory for Infectious Disease Prevention for State Administration of Traditional Chinese Medicine, Tianjin Institute of Respiratory Diseases, Tianjin Haihe Hospital, Tianjin, China; ^3^ Department of Medicine, Division of Pulmonary and Critical Care Medicine, Women’s Guild Lung Institute, Cedars-Sinai Medical Center, Los Angeles, California, United States; ^4^ Clinical Laboratory, Beijing Chao-Yang Hospital, Capital Medical University, Beijing, China; ^5^ Department of Respiratory Medicine, Tianjin Haihe Hospital, Tianjin, China

**Keywords:** AT2 cells, intralobar pulmonary sequestration, endothelial, Tsp1, CD36, Pathology Section

## Abstract

An aberrant systemic artery supply results in recurrent infections in the abnormal lung lobe of intralobar pulmonary sequestration (ILS). The mechanisms underlying such persistent inflammation are unknown. Here, we hypothesize that alteration of an endothelial cell niche for alveolar epithelial cells results in the impaired proliferation potential of alveolar progenitor cells, leading to the defective defense mechanism in intralobar pulmonary sequestration. Paraffin sections of lung tissues from patients with intralobar pulmonary sequestration or from healthy controls were collected for analysis of alveolar epithelial alterations in intralobar pulmonary sequestration by quantitative RT-PCR or immunofluorescent staining. Differential transcripts were identified between human pulmonary artery endothelial cells and human aortic endothelial cells by microarray. Validation of microarray data by quantitative PCR analysis indicated that thrombospondin-1 expression level is low in near-lesion part but high in lesion part of ILS lobe as compared to healthy controls. *In vitro* 3-D matrigel culture was adopted to evaluate the regulation of alveolar progenitor cells by thrombospondin-1 and CD36. We found that the proliferative potential of alveolar type 2 stem/progenitor cells was impaired in intralobar pulmonary sequestration. Mechanistically, we discovered that endothelial thrombospondin-1 promotes alveolar type 2 cell proliferation through the interaction with CD36. These data demonstrate that alveolar stem cells are impaired in the abnormal lobe from patients with intralobar pulmonary sequestration and imply that restoring epithelial integrity can be beneficial for the future treatments of recurrent infections in lung pathologies.

## INTRODUCTION

Intralobar pulmonary sequestration (ILS) is a rare congenital malformation characterized by an abnormal mass of dysplastic lung tissue supplied by an aberrant systemic artery. It accounts for 0.15 to 6.4% of congenital lung malformations. Recurrent infections are the major complication of ILS in the abnormal lung lobe [1]. Because medical treatment options are few and the risk of death increases in adulthood due to massive hemoptysis, surgical resection becomes the treatment of choice [2]. There is a lack of understanding of mechanisms underlying recurrent infections in ILS.

Lung epithelium that covers alveolar space plays a critical role in host defense. Alveolar type 2 (AT2) cells play a vital role in maintaining alveolar structure and host defense against infection. AT2 cells secrete surfactant proteins including SPA, SPB, SPC and SPD, to lower surface tension at the air-liquid interface and thus prevent alveolar collapse at end-expiration [3]. In the mouse, deficiency of any of these surfactant proteins has been demonstrated to result in increased susceptibility to foreign pathogens [4-7]. Additionally, both *in vivo* and *in vitro* studies have indicated that AT2 cells are alveolar stem cells and possess differentiation potential into alveolar type 1 (AT1) cells and thus maintain alveolar integrity [8, 9]. Transplantation of AT2 cells attenuates bleomycin-induced alveolar injury [10]. The altered regulation of AT2 cells, therefore, has a close relationship with distal lung pathologies.

Endothelial cells are anatomically in proximity with multiple epithelial stem/progenitor cells in the lung and have been suggested as a prominent component of stem niche [11-13]. Pulmonary endothelial cell-derived matrix metallopeptidase 14 (MMP14) is required for the growth support of human airway basal cells [11]. Pulmonary capillary endothelial cells are activated by tissue injury and promote alveolar epithelial regeneration [12]. Endothelial thrombospondin-1 (Tsp1) has been proposed to promote the differentiation of bronchioalveolar stem cells into AT2 cells [13]. Mice lacking Tsp1 develop spontaneous pneumonia associated with multiple-lineage epithelial hyperplasia [14]. It remains unknown if Tsp1 is involved in AT2 cell function and associated with epithelial pathologies in the abnormal lobe in ILS.

In the present study, we propose that alteration of endothelial cell niche for lung epithelial cells results in the impaired regeneration potential of alveolar stem/progenitor cells, leading to the defective defense mechanism in ILS. We found that AT2 cells have impaired proliferation potential in ILS, and mechanistically endothelial Tsp1 promotes AT2 proliferation. These data together propose a role of Tsp1 in alveolar epithelial homeostasis and shed new light into the pathogenic nature of persistent inflammation in ILS.

## RESULTS

### Impaired reparative potential of distal epithelial stem cells in the ILS lobe

**Figure 1:**
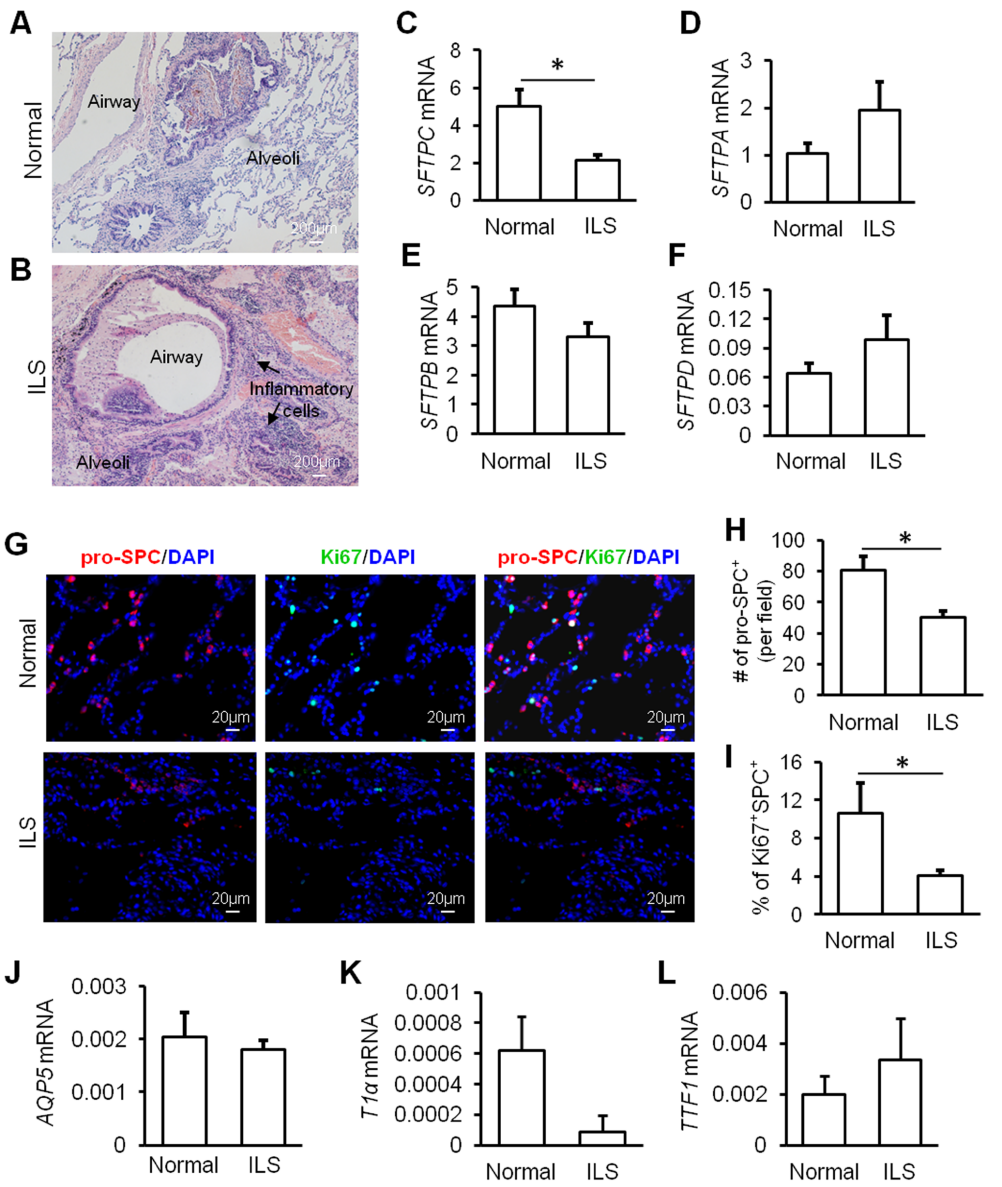
Alveolar homeostasis is disturbed in ILS **A.**-**B.** H&E staining of distal lung tissues from patients with intralobar pulmonary sequestration (ILS) or from health controls. Expression of *SFTPC* mRNA **C.**, *SFTPA* mRNA **D.**, *SFTPB* mRNA **E.**, and *SFTPD* mRNA **F.** were determined with RT-PCR analysis. G-I. Confocal staining of lung tissues with antibodies against SPC and Ki67 indicated a significant loss of SPC^+^ AT2 cells in lesion part of lungs from ILS **G.** This was confirmed by analysis of number of SPC^+^ cells per field (three random fields per section) **H.** Proliferative potential of AT2 cells was assessed by the percentage of Ki67^+^SPC^+^ cells over SPC^+^ cells (*n* = 876 SPC^+^ cells from 3 normal subjects; *n* = 1226 SPC^+^ cells from 3 ILS subjects. 5 regions per subject) **I. J.**-**K.** Expression of *AQP5, T1α* and *TTF1* mRNAs was determined with RT-PCR analysis. *, *P* < 0.05 by unpaired Student *t*-test.

To gain insight into the pathological mechanisms underlying inflammation, we examined abnormal lung lobes that were surgically removed from ILS patients. Examination of paraffin sections of such lobes with H&E staining indicated a large number of inflammatory cells filling the alveolar space (Figure 1A and 1B). Structure of alveoli was severely distorted when compared to that from healthy controls (Figure 1A and 1B). The transcript level of surfactant protein C (*SFTPC*, a specific marker for AT2 cells) was significantly lower in the ILS lobe compared to normal lung (Figure 1C), but levels of *SFTPA mRNA*, *SFTPB mRNA*, and *SFTPD* mRNA were not different between the ILS lobe and normal lung (Figure 1D and 1F). Immunofluorescent staining also indicated that the expression of pro-SPC protein was decreased in AT2 cells (Figure 1G). Furthermore, the number of AT2 cells was reduced by over 40% in the ILS lobe (Figure 1H). Such loss was correlated with reduced proliferation of AT2 cells in the ILS lobe (Figure 1I). Messenger RNA expression of *AQP5* gene, a marker for AT1 cells, remained unchanged (Figure 1J). Another AT1 cell marker T1 alpha (T1α) mRNA expression was decreased but not significantly in ILS (Figure 1K). *TTF1* expression in ILS was similar to that in normal group (Figure 1L). These data suggested that repairing program of alveolar epithelium is altered in ILS.

### Differential gene profiles of HAECs *versus* HPAECs

**Figure 2:**
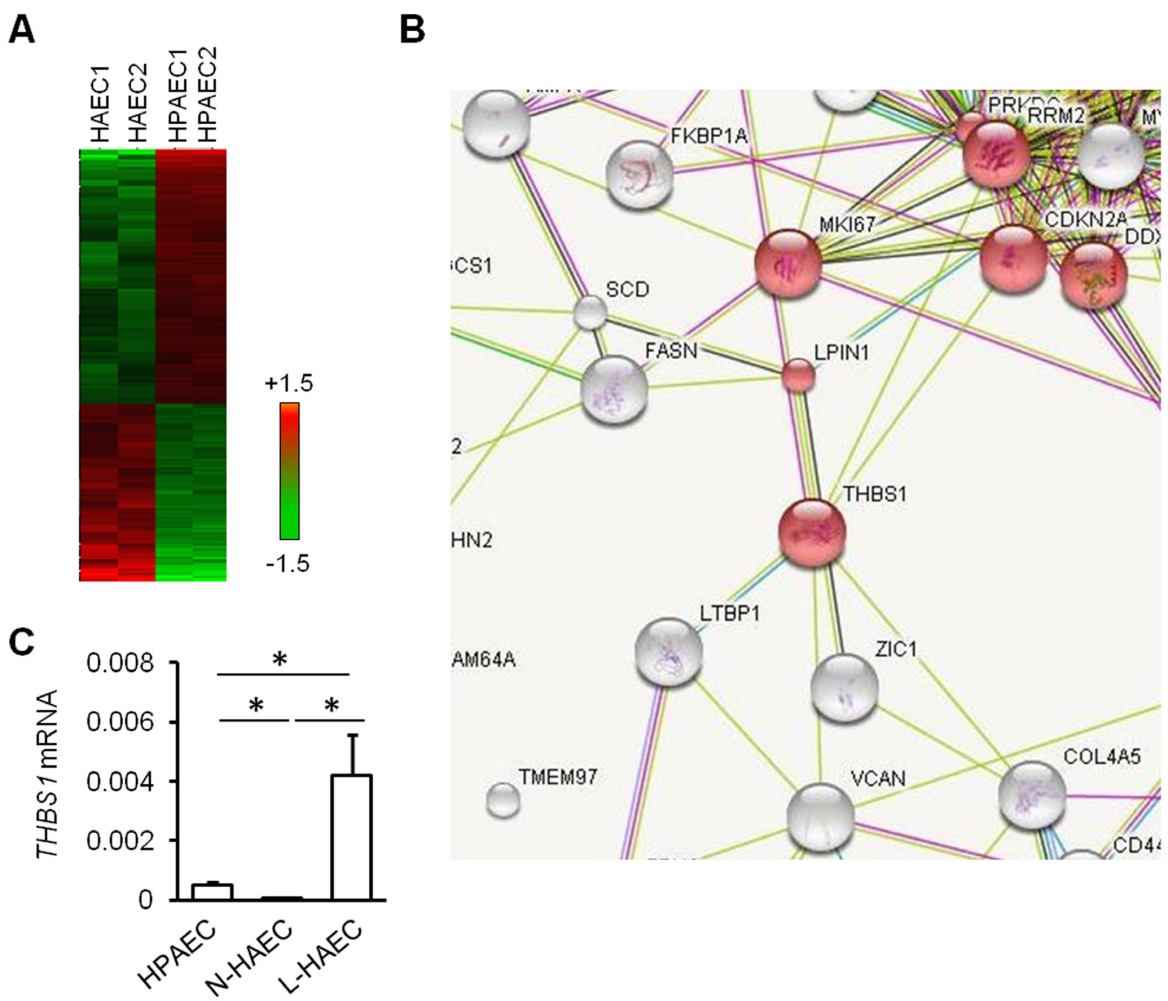
Increased Tsp1 in pulmonary vascular of ILS **A.** Heatmaps indicating differential expression profiles of human aortic endothelial cells (HAEC) *vs*. human pulmonary artery endothelial cells (HPAEC). **B.** STRING analysis shows that Tsp1 has direct connections with 7 differentially expressed genes between HAEC *vs*. HPAEC. **C.** THBS1 (encoding Tsp1) expression was determined in vascular vessel part was dissected from lung paraffin sections from normal subjects, near-lesion region of ILS lobe (N-ILS) and lesion region of ILS lobe. *, *P* < 0.05 by unpaired Student *t*-test.

The arterial supply of the ILS lobe is derived from the aorta or aortic branches instead of the pulmonary artery [15, 16]. To investigate the effects of such alteration to AT2 cells that are in close contact with vascular endothelial cells, we retrieved the expression profile data related to human pulmonary artery endothelial cells (HPAECs) and human aortic endothelial cells (HAECs). Heatmaps were built to show unique gene expression patterns obtained for each cell fraction (Figure 2A). A total of 89 transcripts were enriched in HAEC population, and 185 transcripts in HPAEC population (fold ≥ 2). To illustrate the gene network of biological systems that differs between HAEC and HPAEC, we submitted differential genes, including those enriched in HAEC (fold ≥ 2) and those enriched in HPACE (fold ≥ 2), to the online analysis software DAVID for gene enrichment and functional analysis. The data analysis suggests that HAECs differ from HPAECs in cell division-, chromosome-, phosphoprotein-, cytoskeleton-, nucleotide-binding-, and protein-binding-related features according to the gene enrichment (*P* < 0.001) (Table 1).

**Table 1:** Functional annotation charts of distinct expressed genes between HAEC and HPAEC

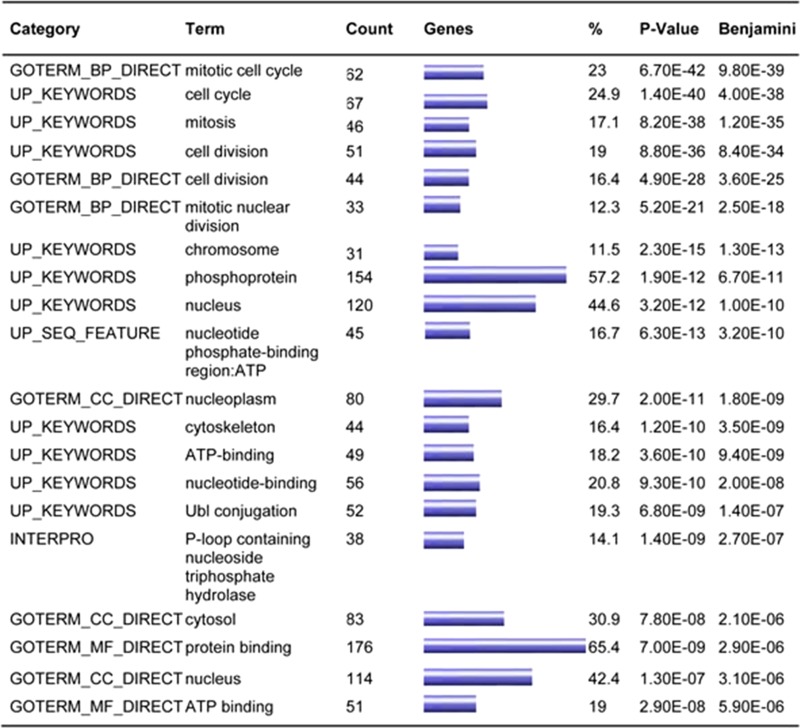

Tsp1 has been demonstrated to be associated with lung infections [14]. To see the connections between Tsp1 and differential genes in HAEC *versus* HPAEC, those 274 significant transcripts were uploaded to the online analysis software “STRING” together with Tsp1. It indicated that *THBS1* gene (encoding Tsp1 protein) had direct connections with 7 differentially expressed genes: *DHFR*, *MK167*, *LPIN1*, *RRM2*, *CDKN2A*, *LTBP1*, and *ZIC1* (Figure 2B).

Both HPAEC and HAEC were not available at hands, we then isolated the vascular part of paraffin-embedded sections of normal lobes from healthy subjects (representing HPAEC), the isolated vascular part of lesion area (representing L-HAEC), and near-lesion area (N-HAEC) of abnormal lobe from ILS subjects to investigate THBS1 expression by quantitative PCR (Figure 2C). Microarray analysis above indicated that *THBS1* expression in HAECs was 75% that in HPAECs (Data not shown). Quantitative PCR validated that *THBS1* mRNA expression was significantly lower in N-HAEC, while it was significantly higher in L-HAEC as compared to HPAEC (Figure 2C). These data suggested that Tsp1 may be involved in lung inflammation associated with ILS.

### Tsp1 promotes mouse AT2 cell proliferation

**Figure 3:**
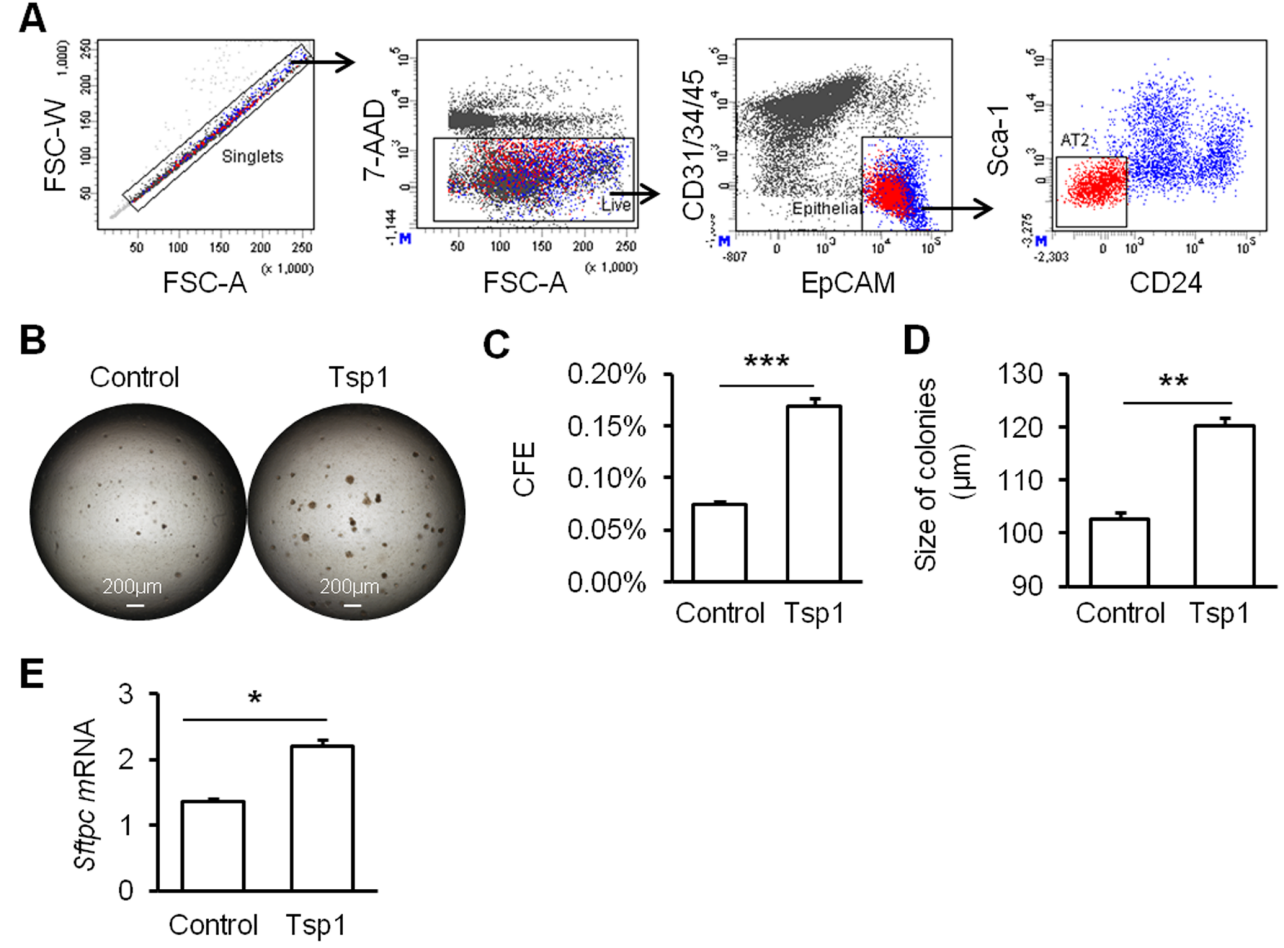
Tsp1 promotes mouse AT2 cell proliferation **A.** FACS strategy of sorting mouse lung AT2 cells. **B.** Matrigel cultures of AT2 cells in the absence or presence of TSP1. **C.** Colony forming efficiency (CFE) of AT2 cells was summarized. **D.** The diameter of epithelial colonies (50μm or greater) was measured and analyzed. **E.** *Sftpc* mRNA expression was determined in matrigel cultures. Three independent experiments were conducted. *, *P* < 0.05, **, *P* < 0.01, ***, *P* < 0.001 by unpaired Student *t*-test.

**Figure 4:**
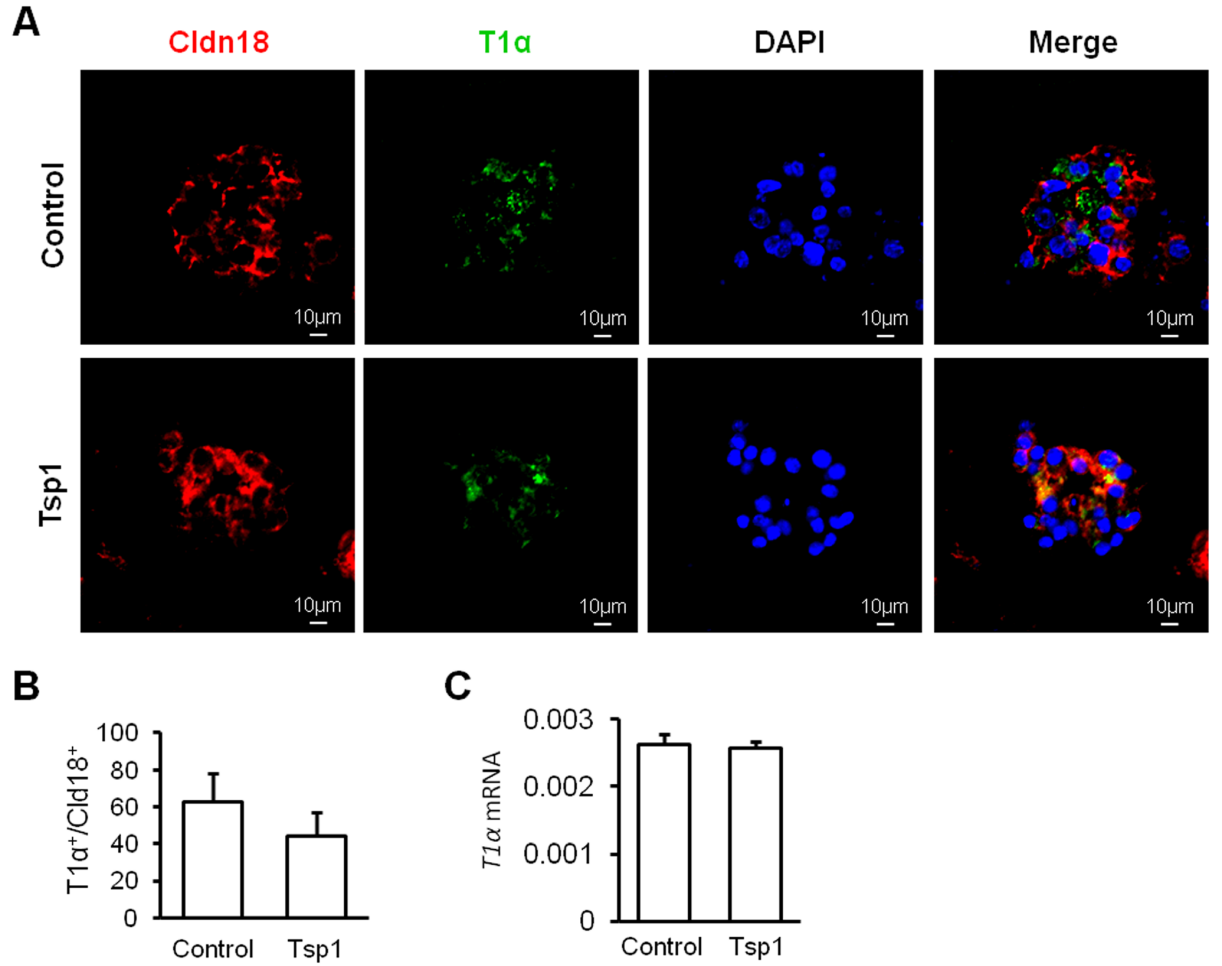
Tsp1 exhibits little effect on mouse AT2 cell differentiation **A.** Representative images of immunostaining of paraffin section of AT2 cell cultures with Claudin 18 (Cldn18) and T1α. **B.** The percentage of T1α^+^ AT1 cells over Cldn18^+^ alveolar epithelial cells from AT2 colonies (*n* = 15) was summarized. **C.** *T1α* mRNA expression was determined in matrigel cultures of mouse AT2 cells. Three independent experiments were conducted.

To investigate role of Tsp1 in alveolar regeneration, mouse AT2 cells were fractionated based on the definition of EpCAM^+^Lin^-^Sca1^-^CD24^-^ (Figure 3A). Mouse AT2 cells were co-cultured with MLg fibroblasts, resulting in the formation of dense and non-lumenal spheres (Figure 3B). Tsp1 was observed to promote the colony forming ability of mouse AT2 cells significantly (Figure 3C). Colony forming efficiency (CFE) of mouse AT2 cells in presence of Tsp1 was two folds that in control group (Figure 3C). To study effects of Tsp1 on AT2 differentiation, we stained the paraffin sections of matrigel cultures of AT2 cells with T1α and claudin 18 (Cldn18, a junction protein and marker for pan- epithelial cells in alveoli) (Figure 4A). By randomly selecting 15 spheres for calculating the number of T1α^+^ AT1 cells and Cldn18^+^ epithelial cells, we observed that the ratio of T1α^+^/Cldn18^+^ was not different between Tsp1 and control group (Figure 4B). And *T1α* mRNA expression in matrigel cultures of AT2 cells was comparable between Tsp1-treated and control groups (Figure 4C). These data suggested that Tsp1 regulates the proliferation, but not the differentiation of mouse AT2 cells.

### Involvement of CD36 in the Tsp1 regulation of mouse AT2 cell proliferation

**Figure 5:**
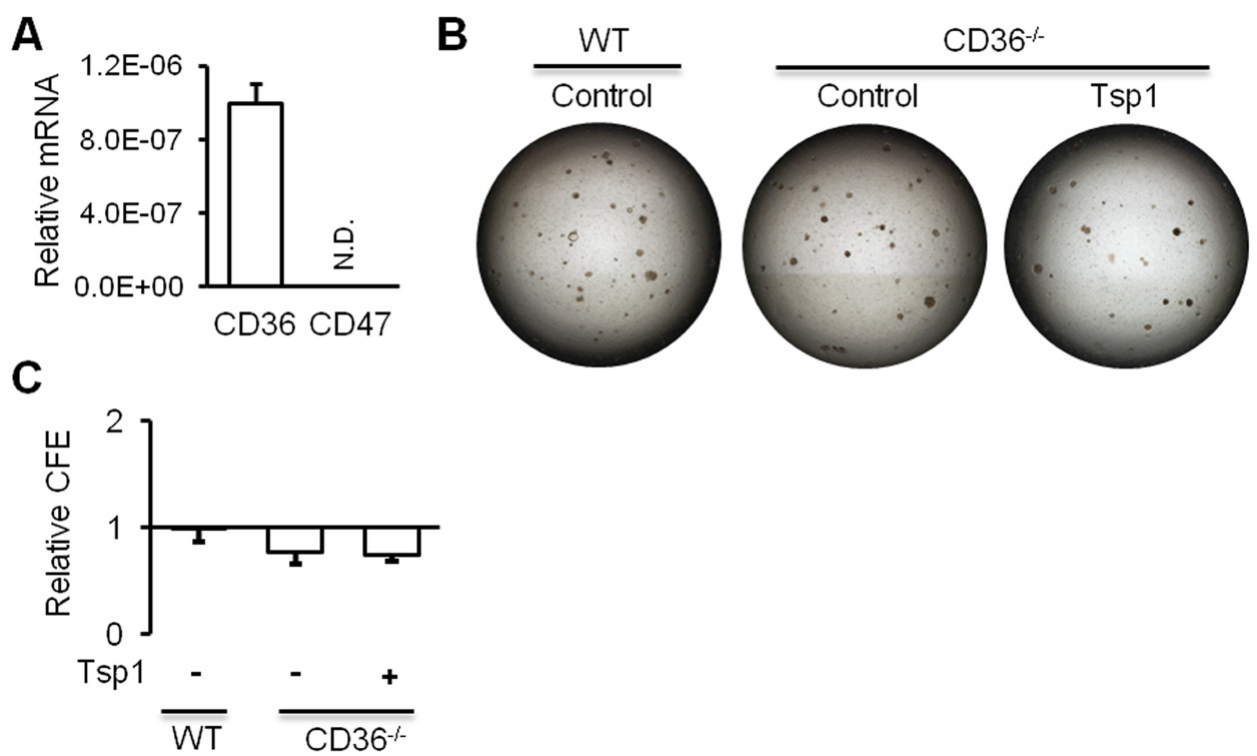
CD36 is required for Tsp1 promotion of mouse AT2 cell proliferation **A.** RT-PCR analysis demonstrated the expression of *CD36* mRNA, but not *CD47* mRNA in mouse AT2 cells. **B.** Matrigel cultures of AT2 cells or CD36^-^ AT2 cells in absence or presence of Tsp1. **C.** Relative colony forming efficiency (CFE) of AT2 cells and CD36^-^ AT2 cells was summarized. Three independent experiments were conducted.

Tsp1 was shown to exert regulatory roles through surface antigens CD36 and CD47 [17]. Quantitative analysis of RNA isolated from sorted AT2 cells indicated that *CD36* mRNA, but not *CD47* mRNA, is expressed in mouse AT2 cells (Figure 5A). To evaluate the role of CD36 in AT2 cell function, AT2 cells were sorted from *Cd36*^*-/-*^ mice and then co-cultured with MLg cells in the presence or absence of Tsp1 (Figure 5B). We observed no difference in CFEs of AT2 cells between cultures with Tsp1 and cultures without Tsp1 (Figure 5C). *Cd36*^*-/-*^ AT2 cells exhibited comparable CFEs to CD36^+/+^ AT2 cells (Figure 5B and 5C). These data suggested that CD36 deficiency abrogated the Tsp1 promotion of mouse AT2 proliferation.

## DISCUSSION

In this report, the centrality of alveolar epithelial stem cells in the inflamed abnormal lung lobe of ILS patients was revealed by stem cell functional studies in human subjects and mice. First, AT2 cells become less proliferative in the abnormal lobe of ILS subjects. Second, validation of gene expression profiles indicated that transcripts including Tsp1 differentiate human pulmonary arterial endothelial cells from human aortic endothelial cells. Third, Tsp1 exhibited no effect on the differentiation of mouse AT2 cells, but promoted their proliferation. Lastly, the regulatory role of Tsp1 on AT2 cell proliferation was abolished in CD36 null mice. Hence, these data support our notion that Tsp1, at least partially through CD36, promotes the regenerative capacity of alveolar stem cells. Low level of Tsp1 in normal aortic endothelial cells fails to maintain alveolar epithelial homeostasis, thus contributing to the prolonged inflammation in the abnormal lobe of the ILS lung.

As secretory cells, AT2 cells secrete surfactant proteins including SPA, SPB, SPC and SPD to minimize the surface tension at the alveolar air-liquid interface, and to clear invaded pathogens [18, 19]. Mice deficient in any of these surfactant proteins become susceptible to infection and injuries [4-7]. Consistent with these data, we observed reduced expression of *SFTPC* mRNA in the abnormal lobe of ILS patients, which may partially explain the susceptibility of such lobe to lung infections for ILS patients. Only *SFTPC* mRNA expression was observed to be reduced in ILS, possibly due to differential source of these surfactant proteins as SFTPB, but not SFTPC, has been reported to be secreted by Club cells as well [20]. It seems that each of these surfactants is regulated differentially and is associated with different lung disorders [3]. As regenerative cells, AT2 cells play a vital role in the maintenance of alveolar epithelium both at steady-state and during the repair process [8, 21]. Mice with AT2 cell failure are prone to die after bleomycin-induced injury [22]. We found that the proliferative potential of AT2 cells is impaired in the abnormal lobe of ILS patients. This finding, coupled with the reduction of *SFTPC* mRNA expression, suggested that repairing capacity is low in ILS lobe, correlating with clinical observation of recurrent lung infections in ILS. It has been demonstrated that Tsp1 enhanced the replenishment of AT2 cells by distal airway epithelial stem cells, which could also relate to the chronic inflammation in ILS [13].

The obvious alteration in ILS is that the abnormal lobe receives an anomalous systemic arterial blood supply instead of a pulmonary vascular supply. Reanalysis of published gene expression profiles in this study pulled out distinct transcripts enriched in HAEC or HPAEC. Pathway analysis indicated that these distinct genes are part of signal pathways associated with cell division, ATP-binding and protein-binding. Endothelial cells derived MMP14 is required for their support of human basal cell growth [11]. Specific deletion of Mmp14 in endothelial cells was shown to impair expansion of AT2 cells after pneuonectomy [23]. Tsp1 null mice exhibited worse alveolar injury after bleomycin treatment [13]. Of note, Tsp1 null mice develop extensive acute and organizing pneumonia spontaneously, and display thickening and ruffling of airway epithelium resulted from increasing cell proliferation [14]. These findings suggest that lack of Tsp1 contributes to the impaired alveolar regeneration. Consistent with this notion, we observed a very low level in near-lesion part of abnormal lobe of ILS. While Tsp1 promotes the proliferation of mouse AT2 cells *in vitro* and such promotion was abolished in CD36 null mice. These data imply that Tsp1/CD36 axis is beneficial to alveolar regeneration. The observation of a high level of Tsp1 in lesion part of ILS reflects the attempt of epithelial repair. We also noticed that lack of CD36 does not lead to reduced proliferation of AT2 cells *in vitro* as expected, which could be due to very low level of its ligand Tsp1 in cultures. But we could not exclude the possibility that other ligands of CD36 including oxidized lipoproteins and fatty acids may play opposite roles in regulating AT2 function [24]. Also, Tsp1 could exert its regulatory roles through binding to integrins, low density lipoprotein receptor-related protein, and a number of other cell membrane-associated proteins [17, 25-27]. In addition, Tsp1 function can be influenced by modification of Tsp1 protein, including glycosylation, which has been shown to be involved in the binding of Tsp1 to CD36 [28]. Considering that Tsp1 can also be expressed by other cell types including platelets and epithelial cells, it is possible Tsp1 derived from these cells could also contribute to alveolar homeostasis.

The mouse study suggested that Tsp1/CD36 regulates the proliferation of AT2 cells. However, other factors could be involved in the alveolar epithelial alteration in the abnormal lobe of ILS. Microarray analysis in this study pulled out the distinct transcripts between HAEC and HPAEC. These distinct genes are possible targets of interest to better understand distal lung pathologies of ILS in the future. As fresh tissues from either normal subject or from ILS patients were not accessible at our hands, we were unable to compare the morphology or quantity of HAEC and HPAEC in those subjects. Besides, the difference in blood pressure between pulmonary artery and aortic artery may play a role too.

In conclusion, our findings suggested that alveolar epithelial stem/progenitor cells exhibited reduced proliferative potential in the inflamed lobe in ILS. The TSP1/CD36 axis between endothelial cells and AT2 cells may play a partial role in the promotion of AT2 cell proliferation. The increased level of Tsp1 in lesion part of ILS lobe indicates repairing attempts in lung. But insufficient repair of distal lung could lead to the susceptibility to lung infections in ILS. Thus, to restore the integrity and functions of epithelial stem/progenitor cells can be promising to treat lung diseases associated with persistent inflammation.

## MATERIALS AND METHODS

### Ethics statement

The use of discarded portions of lung tissue from normal transplant donor falls under Exemption 4 as described in the NIH guidelines. The sample use is under Cedars-Sinai Medical Center approved Institutional Review Board (IRB) (protocol #: Pro00032727), which is approved for the use of biosamples for future research. The use of discarded portions of surgical removed lung tissue from ILS subjects is under the institutional review board of Tianjin Haihe Hospital (protocol #: 2015HHLL02), which is also approved for the use of biosamples for future research.

Mouse experiments were performed according to the protocol approved by Tianjin Haihe Hospital Animal Care and Use Committee (protocol # 2015HHLL03) and in direct accordance with Ministry of Science and Technology of the People’s Republic of China on Animal Care guidelines. All surgeries were performed under anesthesia and all efforts were also made to minimize suffering.

### Human subjects

Independent formalin-fixed, paraffin-embedded (FFPE) tissue blocks of surgically removed distal lung tissue specimens (*n* = 6) were collected from the pathology archive of Tianjin Haihe Hospital. The study was approved by the ethics committee of Tianjin Haihe Hospital (Number: 2015HHLL02). FFPE lung blocks were obtained from discarded portions of normal transplant donor (*n* = 6) lung tissue at Cedars-Sinai Medical Center.

### Mice

C57BL/6 and CD36^-/-^ mice were purchased from the Jackson Laboratory. Mice were housed in pathogen-free conditions and were exposed to a 12-hour light/dark cycle and had free access to food and water. Mice at age 2-4 months were sacrificed for experiments according to the approved protocol (2015HHLL03).

### RNA isolation and real-time PCR analysis

As described previously, RNA was extracted from formalin fixed paraffin embedded (FFPE) tissues [29]. RNA was reverse transcribed with oligo (dT) primer using a SuperScript^®^ III Transcriptor First Strand cDNA Synthesis Kit according to the manufacturer’s instructions. qRT-PCR was conducted with primers (Table 2) using the SYBR Green method on a Roche Light Cycle 96 Real-Time PCR System.

**Table 2:** Sequences of primers for quantitative PCR

Genes	Forward primer	Reverse primer
**Human**		
*β-ACTIN*	5’- GGCACCCAGCACAATGAAGATCAA-3’	5’- ACTCGTCATACTCCTGCTTGCTGA-3’
*SFTPA*	5’- AAGAGCAGTGTGTGGAGATG-3’	5’- CAGAACTCACAGATGGTCAGTC-3’
*SFTPB*	5’- GCATTGCCTACAGGAAGTCT-3’	5’- CCTCCTTGGCCATCTTGTT-3’
*SFTPC*	5’- TTCTTATCGTGGTGGTGGTG-3’	5’- ACCATCTCCGTGTGTTTCTG-3’
*SFTPD*	5’- TGGAGACAAAGGAGCAAAGG-3’	5’- GTGCTGTACTTGTCCCTGTAAG-3’
*AQP5*	5’- GAGCTGATTCTGACCTTCCAG-3’	5’- GAGCAGCCAGTGAAGTAGATT-3’
*T1α*	5’-GATGGAGACACACAGACAACA-3’	5’-CAATGAAGCCGATGGCTAGTA-3’
*TTF1*	5’-CTGGAAGACGATTGGTGAGATG-3’	5’-CTCCAAGCACCACGATTTCT-3’
*TSP1*	5’- AAAGCGTCTTCACCAGAGACCT-3’	5’- GCAGATGGTAACTGAGTTCTGACA-3’
**Mouse**		
*Sftpc*	5’-CTCCTGACGGCCTATAAGCC-3’	5’-TAGTAGAGTGGTAGCTCTCC-3’
*m-T1α*	5’-AAGAGGCATAGAGTCTGGAAATG-3’	5’-ACTGGGCTGGAATGTGTATG-3’
*m-CD36*	5’- ATGACGTGGCAAAGAACAGCAGC-3’	5’- GCAACAAACATCACCACTCCAATCC-3’
*m-CD47*	5’- GTTCAGCTCAACTACTGT-3’	5’- CTCTTATTCGTATGGCTG-3’

### Immunofluorescent staining

Epithelial colonies were fixed in paraformaldehyde, rinsed, immobilized, and embedded in paraffin. Five-micrometer sections were collected and then were incubated with primary antibodies, followed with fluorochrome-conjugated secondary antibody. Slides were mounted and visualized on an Olympus IX73 inverted fluorescent microscope as described previously [30].

### Flow cytometry

As previously described [30], lung single cells were prepared and re-suspended in Hanks’ balanced saline solution buffer, followed by incubating with primary antibodies including CD31-Biotin, CD34-Biotin, CD45-Biotin, CD24-PE, EpCAM-PE-Cy7, and Sca-1-APC. Biotin-conjugated antibodies were detected following incubation with streptavidin-APC-Cy7. Dead cells were discriminated by 7-amino-actinomycin D staining. AT2 cells were then fractionated on a BD FACSAria III sorter.

### Matrigel cultures of AT2 cells

As previously described [30], flow sorted AT2 cells were mixed with mouse fibroblast MLg2908 cells in Matrigel: basic medium (1:1). Cells suspended in Matrigel were added to the chamber of 24-well Transwell filter inserts and placed in 24-well, flat-bottom culture plates containing basic medium with 10 μM SB431542. To test the effect of Tsp1 (Prospec) on the proliferation and differentiation of AT2 cells, culture medium was supplemented with 10 nM Tsp1. Colonies were visualized with an Olympus IX73 inverted fluorescent microscope with a DP80 camera.

### Microarray analysis

Expression profile data in CEL format related to either human pulmonary artery endothelial cells (HPAEC) and human aortic endothelial cells (HAEC) were acquired from the GEO repository (GEO accession: GSE21212), in which HAEC samples (GSM530367 and GSM530369) and HPAEC (GSM530371 and GSM530373) were analyzed using Affymetrix HT Human Genome U133A Arrays. Selected datasets were annotated with Affymetrix Expression Console software. Probe sets that can be used to discriminate HAEC from HPAEC were chosen for further analysis. Gene expression heatmaps were made using Treeview (ver.1.60) software. Signature genes were submitted to the online software ‘‘Database for Annotation, Visualization and Integrated Discovery’’ (DAVID) 6.7 for inquiring functional annotations and gene enrichments [31]. Significant expressed genes were mapped according to their direct or indirect interactions by the web-server STRING 9.0 [32].

### Statistical analysis

Data from three or more independent experiments were collected and analyzed as mean ± SEM. The significance of the results was assessed by an unpaired *t* test between two groups, and one-way or two-way ANOVA followed by post-hoc tests for multiple comparisons. A *P* value < 0.05 was considered significant.
